# Gender and Context Matter: Behavioral and Structural Interventions for People Who Use Alcohol and Other Drugs in Africa

**DOI:** 10.3390/ijerph19148661

**Published:** 2022-07-16

**Authors:** Wendee M. Wechsberg, Isa van der Drift, Brittni N. Howard, Bronwyn Myers, Felicia A. Browne, Courtney Peasant Bonner, Tara Carney, Jacqueline Ndirangu, Yukiko Washio

**Affiliations:** 1 Substance Use, Gender and Applied Research Program, RTI International, Research Triangle Park, NC 27709, USA; ivanderdrift@rti.org (I.v.d.D.); bhoward@rti.org (B.N.H.); fbrowne@rti.org (F.A.B.); cpbonner@rti.org (C.P.B.); jndirangu@rti.org (J.N.); ywashio@rti.org (Y.W.); 2 Gillings School of Global Public Health, University of North Carolina at Chapel Hill, Chapel Hill, NC 27599, USA; 3 Department of Psychology, North Carolina State University, Raleigh, NC 27695, USA; 4 Department of Psychiatry and Behavioral Sciences, Duke University School of Medicine, Durham, NC 27708, USA; 5 Alcohol Tobacco and Other Drug Research Unit, South African Medical Research Council, Cape Town 7580, South Africa; bronwyn.myers-franchi@curtin.edu.au (B.M.); tara.carney@mrc.ac.za (T.C.); 6 Department of Psychiatry and Mental Health, University of Cape Town, Cape Town 7701, South Africa; 7 Curtin enAble Institute, Faculty of Health Sciences, Curtin University, Perth 6102, Australia; 8 Lewis Katz School of Medicine, Temple University, Philadelphia, PA 19122, USA

**Keywords:** alcohol and other drug use, drug use, interventions, women, social determinants

## Abstract

Heavy alcohol consumption and other drug use are prominent across Africa and increase the risk of exposure to violence, HIV acquisition, and other life-threatening injuries. This review synthesizes evidence on alcohol and other drug (AOD) interventions in Africa; evaluates the differences between interventions that do and do not specifically target populations that use AODs; and highlights the impact of comprehensive vs. brief interventions and those that address syndemic issues from a gender and contextualized lens. Literature searches were conducted to identify research outcomes of randomized interventions published between January 2010 and May 2022 that address AOD use in Africa. Thirty-five full-text articles were included in this review. Most of the identified research studies were concentrated in a few countries. Most studies were conducted in South Africa. Many of the studies comprised brief interventions. However, the most comprehensive interventions were the most effective for AOD outcomes. Several studies indicated the importance of addressing AOD use alongside gender-based violence, mental health needs, gender roles, and other social determinants that affect health outcomes. Intervening on AOD use and addressing social determinants from a gender and contextually relevant perspective is essential to ensuring the long-term health and well-being of people in Africa.

## 1. Introduction

Alcohol remains one of the most commonly used substances in the world, and it is often used in combination with other drugs [1,2]. Heavy alcohol consumption and other drug misuse are prevalent across Africa because of the historical cultural norms and social acceptability of binge drinking within patriarchal societies; the increasing popularity of home brewed beer, wine, and flavored spirit coolers with high alcohol content; and the continent’s ties with the drug trafficking trade [3,4,5,6,7]. In addition to alcohol, the main substances used across the African continent include cannabis, methamphetamine, heroin, and other opiates [8,9], which are frequently mixed together or mixed with other harmful chemicals, such as battery fluid, for a stronger high [6,10].

Recent supply chain disruptions and restrictions on the sale and distribution of alcohol associated with the COVID-19 pandemic have led to a further surge in the use and mixing of alcohol and other drugs (AODs), an increase in gender-based violence (GBV), and a significant decrease in the number of persons accessing HIV and other medical treatment because of pandemic-related closures across all African regions [3,11,12]. AOD use can increase the risks of mental health issues, adverse sexual and reproductive health (SRH) outcomes, exposure to violence, social and economic instability, road accidents, suicide, and other life-threatening injuries [13,14,15]. This is especially concerning for young people under the age of 25 using AODs because it compromises brain development and consequently growth, and limits their opportunities to reach their full potential at a critical developmental period [16,17]. The burden of disease associated with alcohol consumption in Africa is expected to rise because of a growing population of young people—an “emerging market” and prime target group for multinational alcohol corporations—consumption in sub-Saharan Africa increases as much as 150% in this group [5,8,14].

Social determinants and contextual factors such as poverty, education level, economic status, housing, discrimination, and social stigma remain critical factors in determining who is most at risk for or affected by AOD use—such as women, girls, and youth, who often experience an interwoven burden of social and health consequences, including heightened risks for GBV, HIV, trauma, and discrimination from the community or healthcare providers [13,18,19]. AOD use among women intersects with increased risk for HIV and GBV [20,21], both of which are prevalent in African countries with high AOD misuse. For example, South Africa has the highest prevalence of fetal alcohol spectrum disorders (FASD), the highest number of women living with HIV, and highest GBV statistics globally [22,23,24]. AOD use significantly increases the likelihood of women’s exposure to intimate partner violence (IPV) because it limits self-awareness, personal power, and control for deescalating situations [25,26,27].

AOD use also affects people living with HIV (PLWH) by reducing adherence to antiretroviral therapy (ART) [28,29,30]. New multifaceted approaches have helped to reduce HIV across Africa: Botswana, Eswatini, and Namibia having achieved the UNAIDS 90-90-90 HIV care cascade targets [31]. However, AOD use may temper the potential of these advances, as the number of PLWH in East and Southern Africa is still increasing among key populations, even as HIV testing and ART are becoming more accessible [11,31].

Collectively, research suggests that AOD use contributes to adverse outcomes across the lifespan of key populations. Alcohol is a primary substance of use across Africa with dire consequences [22]. Interventions to reduce AOD use among key populations in Africa have been developed and tested in trials, and previous reviews of their effectiveness have been conducted [32,33,34,35,36]. However, to the best of our knowledge, no reviews have synthesized evidence in support of their effectiveness while also examining the impact of culturally adapted and gender-specific interventions. The present review begins to address this gap. More specifically, this review synthesizes the findings from randomized controlled trials (RCTs) of AOD interventions in Africa and categorizes interventions that focus specifically on AOD use as a primary outcome and those that focus on AOD use as a secondary outcome. Additionally, this review examines the impact of culturally tailored, gender- and context-specific interventions on AOD use and health outcomes. This review expands previous reviews by looking at the intersectionality of gender and context.

## 2. Methods

**Figure 1:**
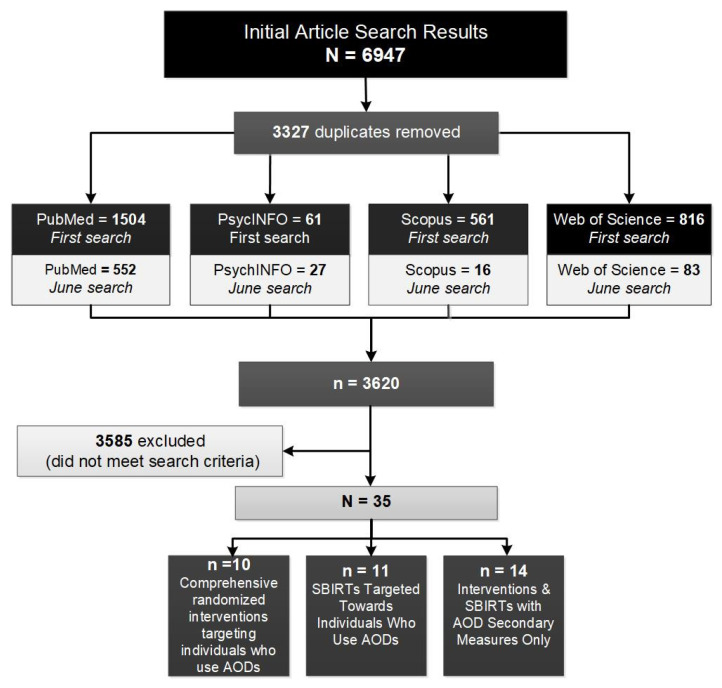
Search Methods.

Initial literature searches were conducted between July and October 2021, followed by a second search conducted in June 2022, to identify outcome studies for interventions that address AOD use in Africa. PubMed, Web of Science, Scopus, and PsycINFO were searched, returning 2942 results, and 678 total unique results in each search (see Figure 1) (see search terms in Appendix A). A supplementary search in Web of Science was conducted to determine whether expanded search terms would yield more relevant results; however, after reviewing the additional results, we determined that secondary searches of all databases were not necessary. We used Boolean search terms to identify relevant publications that met the following criteria: (1) published between January 2010 and October 2021, and a second search was conducted to include any publications from October 2021 through May 2022 (since the original review); (2) reported the findings of interventions that either specifically addressed or measured AOD use as outcomes; (3) reported the findings of interventions that were conducted in the continent of Africa; and (4) written in English. We downloaded the results into an EndNote library for further review and used a priori criteria to review publications. Publications were sorted by using a categorical folder system, where articles were labeled as “Yes,” “No,” or “Maybe,” through an initial review of publication abstracts. Conference abstracts, dissertations, theses, and book chapters were excluded from the initial search. Two trained researchers reviewed the full text of articles labeled “Maybe” or “Yes” to determine whether they met all inclusion criteria. Publications were also evaluated for study design, power, and comprehensiveness to determine study rigor.

Of the 4612 abstracts returned in the initial search, and 2335 in the second search, 1670 duplicates across databases were removed in the first search and 1657 were removed in the second search; then, 2942 were excluded because they were not relevant to this review in the first search, and 676 were excluded in the second search. Ultimately, 35 full-text articles were included in this review. Several relevant protocol [37,38,39,40,41,42,43,44,45,46,47], baseline or pilot [48,49,50,51,52,53,54,55], and feasibility studies [56,57,58,59,60,61,62,63,64,65,66,67,68,69] were identified; however, they did not meet the inclusion criteria because trial outcomes were the focus of this review.

We categorized the results as follows:

− **Comprehensive interventions targeting individuals who use AODs**, including randomized trials with at least two arms (including a control arm), a sample size of at least 100 participants, and study eligibility criterion of AOD use. These interventions address AODs and other issues—such as HIV or GBV—to distinguish them from brief intervention studies that focus on AOD only.
− **Single-session or screening brief intervention and referral to treatment (SBIRTs) targeting individuals who use AODs**, including studies with single or brief intervention (up to four) sessions, with a study eligibility criterion of AOD use.
− **SBIRTs with AOD use as secondary measures**; that is, AOD use was not an eligibility requirement for participants.

## 3. Results

### 3.1. Comprehensive Interventions Targeting Individuals Who Use Alcohol and Other Drugs

Ten of the identified studies evaluated the effectiveness of interventions targeting individuals who used AODs [28,70,71,72,73,74,75,76,77,78], all of which were behaviorally or biobehaviorally focused interventions (see Table 1). The sample sizes of these studies ranged from 124 to 818. All interventions targeted adults (aged 18 or older).

#### 3.1.1. Delivery Setting

Five of the interventions were conducted in South Africa; two in Kenya; and one each in Zimbabwe, Zambia, and Nigeria. Five interventions were conducted with PLWH or people at increased risk for HIV and two interventions were conducted with women who conduct sex work. Four interventions were delivered by trained nurses, lay counselors, or clinic staff at clinic or community health facility sites, including a Motivational Interviewing (MI) intervention via flipchart in one-on-one sessions lasting 20 min on average [70]; a 10-session intervention lasting 45 to 60 min per session was delivered by trained nurses [71]; a two session gender-stratified intervention with an additional spousal support session was delivered by lay counselors [72]; and a six-session intervention with weekly 90 min group sessions was delivered by trained paraprofessional counselors [74]. One implementation science study, which occurred over four stepped-wedge cycles, was delivered by trained clinic staff and researchers in groups at clinics and substance use rehabilitation and treatment centers [28]. One intervention comprised 20 biweekly sessions lasting 50 min each in community secondary school sites [73]. Finally, four interventions were delivered at project field sites or other private community settings by trained interventionists from the target community, including a two-session group intervention delivered by experienced, multilingual female interventionists [75], a 3-h workshop delivered by peer educators in groups of couple dyads [76], a two-session peer-facilitated group intervention with four modules each [77], and a two-session group intervention delivered by trained community interventionists during 50-min sessions [78].

#### 3.1.2. Gender

Five interventions focused on women and two focused on heterosexual couples. The remainder of the interventions were not gender specific. Five interventions used gender-sensitive and empowerment-driven approaches, including skill-building activities and role-playing and rehearsal to reduce AOD use, sexual risk, and violence among women and couples [28,75,76,77,78]. One study in Kenya used culturally adapted gender stratification to avoid reinforcing the secondary status of women [74]. A two-session, gender-stratified intervention with an additional substance use support session for spousal support addressed IPV and interrelated AOD use [72]. This study also used counselors in male–female pairs to recruit heterosexual couples in which the woman reported experiencing recent IPV and measured gender norms as a secondary outcome [72].

#### 3.1.3. Context and Culture

Many studies conducted formative work to inform cultural adaptation of the intervention, including adapting images and content depicting motivational readiness for change, real-life risk scenarios regarding victimization, and AOD use relevant to women who conduct sex work in Kenya and South Africa [70,78]. Another intervention was adapted to include focus group participants’ voices within the intervention for couples in Cape Town and risks associated with drinking at local venues [76], and another for South African women living with HIV [28]. Several interventions were translated from English into local languages [28,75]. One intervention adapted a cognitive behavioral therapy (CBT) intervention [74] by referencing local Kenyan settings, using rural images in treatment materials, addressing culturally prominent misinformation about alcohol, and delivering the intervention in Kiswahili, the official language of Kenya.

#### 3.1.4. Other Intersectional Issues

Five South African studies addressed intersectional issues of AOD use, GBV/IPV, and HIV [28,75,76,77,78], with some incorporating components on overcoming barriers to pre-exposure prophylaxis (PrEP)/ART adherence [28] and healthy communication [76]. A Zambian study jointly addressed AOD, HIV and ART adherence [72]. Two interventions were grounded in CBT, including one with outpatients living with HIV focused on HIV and alcohol education, alcohol abstinence, coping with substance use triggers, problem solving, and refusal skills [74]; and another with heterosexual couples that used a joint cognitive–behavioral, multi-problem, transdiagnostic Common Elements Treatment Approach (CETA) modified to address IPV and alcohol/substance use, partner communication, and related mental health comorbidities [72]. One study used rational emotive health therapy treatment for alcohol use disorder (AUD) symptoms among PLWH, and addressed problematic beliefs related to AUD and practical techniques to reduce symptoms [73]. Another study addressed AOD and HIV risk among women at risk for victimization [70].

### 3.2. Single-Session or Screening Brief Intervention and Referral to Treatment Targeting Individuals Who Use Alcohol and Other Drugs

Eleven studies evaluated the effectiveness of SBIRT approaches for individuals who used alcohol or other substances as primary measures; all were behaviorally focused (see Table 2). These interventions largely had components of MI. Some combined strategies, including blending MI with problem-solving therapy, providing participants with referral and resource lists, or testing multiple intervention types together to determine their effectiveness.

#### 3.2.1. Delivery Setting

Nine interventions were conducted in South Africa, and there was one intervention each in Uganda and Kenya. Ten interventions were delivered in an individual setting [79,80,81,82,83,84,85,86,87,88] and one intervention in a group setting [89]. All interventions were delivered at health clinics or study field sites. Seven interventions were delivered by trained nurses and counselors, two interventions by lay counselors recruited from the community, and one intervention by trained research staff. The interventions included components of MI and problem-solving therapy [79,80,82,83,85,86,87], substance use reduction strategies [84,85,89], comprehensive assessments of a participant’s alcohol use [86], standardized positive prevention counseling [83], referrals and resources [87], and brief counseling sessions on alcohol risk reduction [88].

#### 3.2.2. Gender

Only two interventions were gender-specific, with both focusing on women who were pregnant or women at risk of an alcohol-exposed pregnancy [82,86]. One of these interventions focused on SRH to promote birth control uptake and reduce risky substance use to prevent risk for alcohol-exposed pregnancies.

#### 3.2.3. Other Intersectional Issues

Two interventions addressed either HIV or tuberculosis (TB) and aimed to improve treatment outcomes by addressing risky AOD use [83,88]. Two interventions also considered mental health promotion strategies [79,84]. One study addressed HIV and AOD use using the Information–Motivation–Behavioral Skills (IMB) model and personalized Alcohol Use Disorders Identification Test (AUDIT) feedback [81].

However, no interventions addressed additional contextual factors, such as GBV/IPV, although the role of AOD use on these issues was acknowledged.

**Table 2:** Single-session or screening brief intervention and referral to treatment (SBIRTs) targeting individuals who use alcohol and other drugs.

Citation	Region/Sample	Setting/Topics	Intervention	Outcomes
Behavioral				
Harder et al. (2020) [80]	**Kenya:** 300 adults with alcohol use problems	Health center staff administered the in-person intervention (one session, 30 min) or spoke with health center staff by telephone for those in the mHealth condition EtOH	Arm 1: mHealth MI delivered via telephone Arm 2: MI delivered in person	No differences between mHealth or in-person MI, but participants in the waiting list control group had higher AUDIT-C scores than participants in intervention arms
Huis in’t Veld et al. (2019) [81]	**South Africa**: 560 adults living with HIV	Four nurses not associated with the clinics delivered the interventions to patients and completed the follow-ups at clinic sites EtOH, HIV	WHO brief intervention package for harmful drinking: advice on AUDIT scores, health education leaflet, and brief counseling	* AUDIT scores decreased in both groups compared with baseline, but no significant impact over time No between-group differences
Marais et al. (2011) [86]	**South Africa**: 194 women who were less than 20 weeks pregnant and more than 15 years of age (rationale for sampling was to destigmatize FASD)	Brief interventions varying in length and objective delivered individually by trained staff EtOH	A series of four brief interventions and a comprehensive assessment addressing alcohol use among pregnant women	* Decrease in AUDIT between arms
Mertens et al. (2014) [85]	**South Africa:** 403 young people aged 18 to 24 who use AOD seeking primary care at a clinic	Individual session in a large public-sector primary health clinic in Delft, Cape Town, delivered by a trained nurse to address alcohol and substance use among young people EtOH, THC, METH, Methaqualone, Sedatives	A brief motivational intervention and a referral resource list for drinking and drug use	* Reductions in ASSIST alcohol involvement scores larger for the intervention condition
Peltzer et al. (2013) [88]	**South Africa**: 1196 participants from selected clinics with active tuberculosis who scored less than 7 or 8 on the AUDIT (women/men)	Lay counselor delivered individual two-session intervention TB, EtOH	Brief counseling sessions on alcohol risk reduction	Reductions in AUDIT score, hazardous or harmful drinking, alcohol dependence, heavy episodic drinking, and hazardous drinking in treatment groups
Pengpid et al. (2013) [89]	**South Africa:** 392 outpatients who were screened for alcohol problems	Intervention delivered by the intervention assistant nurse counselor in groups at a hospital in Tshwane EtOH	Brief intervention on substance use strategies for reduction, including sensible limits, diaries, problem solving, and goal setting	No significant intervention effects on alcohol use, including AUDIT score
Pengpid et al. (2013) [84]	**South Africa**: 152 university students who scored as risk drinkers through the AUDIT	One 20-min intervention session delivered by counselors EtOH, THC, MH	Brief intervention addressing AUDIT score results and providing education and strategies for reducing risky drinking	No significant between-group changes in AUDIT score, but reductions in all treatment groups over time
Rendall-Mkosi et al. (2013) [82]	**South Africa**: 196 women engaging in risky drinking with ineffective or no contraceptive use	Five-session MI delivered individually by locally recruited and trained lay counselors at study sites EtOH, THC, SRH	MI focused on supporting behavior change based on the Project CHOICES study along with an information pamphlet about FASD and a women’s health handbook	* 50% reduction in the proportion of women at risk for an alcohol-exposed pregnancy in the MI group * Greater reduction in the proportion of women not using birth control in the MI group Reduction in women who met the criteria for risky drinking in both groups
Sorsdahl et al. (2015) [79]	**South Africa**: 335 patients at emergency departments in health clinics at moderate to high risk for substance use problems based on the ASSIST	MI arm: 20-min intervention delivered individually by a counselor at a community health center MI + PST arm: Five 45- to 60-min sessions delivered individually by a counselor at a community health center EtOH, THC, COC, METH, Methaqualone, MH	MI: adapted from the ASSIST-Linked Brief Intervention MI + PST: multiple interventions building goals for substance use reduction and learning techniques of PST	* ASSIST scores lower in MI + PST group than MI and control No significant difference between the MI and control groups * Lower CES-D score in MI + PST group compared with the MI and control groups No differences in AOD-related injury, violence, or police interaction between groups
Wandera et al. (2017) [83]	**Uganda**: 337 adults living with HIV that use alcohol (scored less than or equal to 3 on AUDIT-C)	Standardized positive prevention counseling and MI counseling delivered by trained counselors alone at the study site EtOH, HIV	Standardized positive prevention counseling plus MI counseling	* Reduction in mean AUDIT-C score at 6-month follow-up in both arms * Women had a greater AUDIT-C reduction in the MI group than in the control group No between-group differences
Ward et al. (2015) [87]	**South Africa**: 403 young adults aged 18 to 24 who use AODs	Trained nurse practitioners delivered the intervention individually EtOH, THC, Methaqualone, COC, METH, Sedatives	Brief motivational intervention and a referral resource list	No significant between-group differences * Observed that those who reduced alcohol consumption also reported reduced aggressive behaviors

**Abbreviations:** AMP = amphetamines; AOD = alcohol and other drugs; ASSIST = Alcohol, Smoking and Substance Involvement Screening Test; AUDIT = Alcohol Use Disorders Identification Test; CBT = cognitive behavioral therapy; CES-D = Center for Epidemiologic Studies Depression Scale; CETA = Common Elements Treatment Approach; COC = cocaine; EtOH = alcohol; FASD = Fetal alcohol Spectrum Disorders; GBV = gender-based violence; IPV = intimate partner violence; METH = methamphetamines; MH = mental health; mHealth = mobile health; MI = Motivational Interviewing; MI + PST = Motivational Interviewing + Problem Solving Therapy; OPI = opiates; SRH = sexual and reproductive health; TB = tuberculosis; THC = cannabis; WHO = World Health Organization. * Denotes significant finding.

### 3.3. Interventions and SBIRTs with AOD Use as Secondary Measures

Fourteen studies reported on AOD use outcome measures of interventions that did not directly target AOD (see Table 3). Eight interventions were behaviorally focused interventions, two interventions were behavioral-structural interventions, and four interventions were SBIRTs.

#### 3.3.1. Delivery Settings

Nine of the studies were conducted in South Africa; one multisite study was conducted across Tanzania, Kenya, and Namibia; and one study each was conducted in Kenya, Zimbabwe, Nigeria, and Uganda. Four interventions were delivered individually, and eight interventions were delivered in group settings. Six interventions were delivered by peer educators, community members, or lay counselors; and eight interventions were delivered by professionals, including educators, counselors, dieticians, and healthcare providers. These interventions enrolled women who were pregnant, men, adolescents, individuals at increased risk for or living with HIV, employees at a safety and security company, members of a market traders association, parents or caretakers of adolescents, individuals receiving treatment for pulmonary TB, and adults from prioritized communities. Intervention types included home visits [90], brief and motivational interventions [91,92,93], six 75 min intervention group sessions [94], a school-based intervention [95], four 3-h intervention sessions [96], financial incentives [97], a parenting skills intervention [98], a lifestyle behavioral intervention [99], a clinic-based intervention [100], a training workplace-based intervention [101], and a community-based structural–behavioral intervention [102]. Study samples ranged from 185 to 11,448 participants.

#### 3.3.2. Gender and Culture

One intervention focused on women, and three interventions focused on men, one of which engaged men in conversations about masculinity and “responsible manhood.” The woman-focused intervention used home visits to promote maternal health and monitor mental health needs and AOD consumption [90]. One intervention focused on men used same-gender community facilitators to discuss HIV risk-reduction and condom use, and increase HIV/STI knowledge [94]; another intervention promoted healthy masculinity and reducing violence among rural and peri-urban men [96]; and another intervention focused on men and evaluated the effectiveness of financial incentives in reducing spending on alcohol, gambling, and transactional sex [97].

#### 3.3.3. Other Intersectional Issues

Four interventions addressed SRH, explaining and addressing the ways that AOD use can lead to risky sexual behavior. One intervention addressed IPV among women who were pregnant [90]; another intervention addressed IPV related to HIV disclosure [103]. One intervention also jointly addressed mental health through dialogue and monitoring pregnant and postpartum women in home visits [90], and one intervention jointly addressed gambling and AOD use through financial incentives promoting healthy behaviors [97] and employee wellness [101]. One intervention focused on parenting skills and reducing violence between parents and their adolescent children. Another intervention focused on adherence to TB treatment. Another intervention that focused on nutrition and hypertension also addressed excessive alcohol use through a lifestyle and behavioral interventions that incorporated physical fitness promotion and health knowledge [99].

Many of the interventions that used AOD use as a secondary measure did so to promote adherence to HIV treatment or HIV prevention outcomes. One intervention aimed to reduce violence related to HIV disclosure [103], whereas others were HIV risk-reduction interventions [94,102]. Two interventions worked directly with individuals living with HIV [91,100] (see Table 3).

**Table 3:** Interventions and single-session or screening brief intervention and referral to treatment (SBIRTs) with alcohol and other drug use as secondary measures.

Citation	Region/Sample	Setting/Topics	Intervention	Outcomes
Behavioral				
Burnhams et al. (2015) [101]	**South Africa:** 325 safety and security division employees of a municipality	Six training modules presented to employees in groups over an 8-h session delivered in the workplace by interventionists EtOH	TA: evidence-based workplace training program that addresses behavioral risks and stigma and promotes help-seeking and proactive behaviors	* TA had the greatest impact on days having 5 or more drinks at one sitting in the past 30 days * TA showed modest reductions in binge drinking from baseline to 3-month follow-up and going to work or calling in sick because of a hangover
Eze et al. (2020) [99]	**Nigeria:** 376 adults who were registered members of the market traders’ association	Public health physician, dietician, and physical fitness counselor facilitated group intervention over two sessions of 5-h each EtOH, Nutrition	A lifestyle and behavioral modification intervention to control hypertension by promoting increased physical activity and dietary adjustments	* At post-test, intervention participants reduced excessive alcohol consumption and increased physical activity and fruit and vegetable servings, resulting in overall lower risk for hypertension
Jemmott et al. (2014) [94]	**South Africa:** 1181 men from 22 geographical neighborhood clusters	Six 75-min group intervention modules facilitated by men from the community at the University of Fort Hare in East London HIV, EtOH	HIV risk-reduction intervention to improve condom use and increase HIV/STI knowledge	* Intervention effects were increases in consistent condom use, talking to partners about condom use, and frequency of condom use
Manyaapelo et al. (2019) [96]	**South Africa:** 575 young men aged 18 to 35	Four 3-h group sessions delivered by peer educators over 4 weeks AOD, SRH	Ubudoda Abukhulelwa Responsible Manhood: program developed for soon-to-be-released justice-involved men addressing masculinity, sexual relationships, communication, and AOD use	* Increases in intentions to reduce AOD use and changes in attitudes toward avoiding sex when one is intoxicated among men in the intervention
Massarwi et al. (2021) [98]	**South Africa**: 525 parents and caregivers of adolescents	Fourteen-session parenting program lasting 1 to 1.5-h per week AOD, MH	Parenting for Lifelong Health/Sinovuyo Teen: program to promote family cohesion and nonviolent discipline, improve parent-child relationships, and improve communication in low-resource settings	* Parental substance use reduction was associated with a reduction in parental depression at 5- and 9-month follow-ups
Moscoe et al. (2019) [97]	**Kenya:** 300 men aged 21 or older engaged in fishing or transportation work	Provided financial incentives based on the amount of savings in a registered bank account EtOH, Gambling	Participants received weekly financial rewards if they saved money by not spending on alcohol or gambling	The intervention group had higher growth in bank saving balance but no differences in spending on alcohol or gambling between groups
Rotheram-Borus et al.(2015) [90]	**South Africa:** 1238 pregnant women residing in urban, low-income neighborhoods in Cape Town, South Africa	Home visits by community health workers EtOH, IPV, MH	Home visits promoting educational knowledge and behavior change on HIV/TB, alcohol, mental health, breastfeeding, and malnutrition	* Mothers in the intervention arm were less likely to report depressive symptoms and more likely to report positive quality of life at 36-month follow-up * Drinking increased over the 5 years post-birth, but participants in the intervention arm had smaller increases
Tibbits et al. (2011) [95]	**South Africa:** 4040 youth attending nine schools in Mitchell’s Plain, Cape Town	Teacher-delivered intervention over two to three class periods in groups EtOH, THC, SRH	HW is a school-based intervention promoting social-emotional skills, substance use and sexual behavior knowledge and refusal skills, and healthy free-time activities	* Greater reduction in rate of polydrug use in women and frequent polydrug use among participants in HW Reduction in lifetime sexual activity and refusal to have condomless sex in the HW arm
**Combination Behavioral and Structural**				
Bachanas et al. (2016) [100]	**Namibia, Kenya, and Tanzania:** 3522 patients living with HIV attending clinical care	Clinic-based package of HIV prevention interventions delivered in 9 of 18 clinics by health care providers in groups EtOH, SRH	HIV prevention intervention provided by trained clinicians and lay counselors	* Sexual barrier use outcomes achieved by the community health care staff were comparable to or better than those achieved by the Partner Project research staff, and both were superior to the control group * A reduction in IPV was observed for the entire sample, although no change in alcohol use was observed
Cubbins et al. (2012) [102]	**Zimbabwe:** 185 individuals aged 18 to 30 living within 30 selected sample sites	CPOLs shared intervention messages in social and community settings EtOH, HIV	60 CPOLs trained to deliver HIV risk- reduction messages, with not drinking alcohol being a secondary message	Community-level analyses found no differences between groups * Declines in alcohol use, frequency of use, and quantity of drinks were found in intervention and control sites at relatively equal levels
**SBIRTs, Motivational Interviewing**				
Louwagie et al.(2022) [93]	**South Africa**: 574 adults beginning treatment for drug-sensitive pulmonary TB	Lay health workers delivering three MI sessions along with SMS messages to bolster intervention content EtOH, TB	ProLife Intervention: Participants created plans to address alcohol and tobacco use and TB adherence followed by 10 SMS messages supporting TB treatment adherence	Reductions in AUDIT scores at follow-up but no significant intervention effect
Peltzer et al. (2010) [91]	**South Africa**: 488 adults living with HIV receiving services at HCT clinics	Three sessions (20 to 30 min each) delivered by lay counselors individually EtOH, HIV	Motivational skills-building risk reduction counseling intervention assisting individuals living with HIV to reduce sexual risk behaviors and alcohol consumption	* Reductions in drinking and various sexual risk behavior following intervention
Pitpitan et al. (2015) [92]	**South Africa**: 617 participants from an STI clinic in Cape Town, South Africa	Group counseling and intervention providing education EtOH, SRH	Brief risk-reduction intervention about HIV transmission and risk behaviors, including alcohol use as a risk factor	* IMB model-based intervention reduced alcohol risk behaviors and expectancies
Wagman et al. (2015) [103]	**Uganda:** 11,448 individuals aged 15 to 49 from preexisting clusters who agreed to provide blood samples for HIV testing	Surveys conducted by same-sex interviewers in private; intervention conducted in groups by RHSP counselors at project site EtOH, IPV, HIV	S.H.A.R.E. violence reduction intervention: consisted of screening and a brief intervention to reduce IPV related to HIV disclosure and address risk behaviors	* Proportion of women experiencing IPV lower among intervention participants * Women’s rates of HIV disclosure were higher in the intervention group * Lower HIV incidence among men in the intervention group

**Abbreviations:** AMP = amphetamines; AOD = alcohol and other drugs; AUDIT = Alcohol Use Disorders Identification Test; CBT = cognitive behavioral therapy; CES-D = Center for Epidemiologic Studies Depression Scale; CETA = Common Elements Treatment Approach; COC = cocaine; CPOLs = Community Popular Opinion Leaders; EtOH = alcohol; FASD = Fetal alcohol Spectrum Disorders; GBV = gender-based violence; IMB = Information–Motivation–Behavioral; IPV = intimate partner violence; METH = methamphetamines; mHealth = mobile health; MH = mental health; MI = Motivational Interviewing; OPI = opiates; RHSP = Rakai Health Sciences Program; SHARE = Safe Homes and Respect for Everyone; SMS = Short Message Service; SRH = sexual and reproductive health; STI = sexually transmitted infection; TA = Team Awareness; TB = tuberculosis; THC = cannabis; WHO = World Health Organization. * Denotes significant finding.

## 4. Discussion

This review aimed to present an overview of the research and the commitment of researchers to the issue of AOD use with gender and context in mind in Africa. However, only a few countries were represented, possibly because our review was limited to studies written in English and randomized trials; also, studies with tobacco only were not included. Sixty-three percent of the reported interventions were conducted in South Africa. In South Africa, especially the Western Cape, there are numerous winelands and a large alcohol industry that in the past has paid farmworkers wages with alcohol [104], which contributed to alcohol use for generations [105,106] that continues today [107]. Studies also indicated various drug use, which often changes over time and across regions. South Africa had greater methamphetamine and amphetamine type drug use; and more recently, methaqualone mixed with cannabis. Nonetheless, alcohol either as the primary or secondary outcome, was the major drug reported. Consequently, a focus on key populations who use AODs in areas of scarce resources and underemployment is essential. Several studies highlighted the importance not only of individual differences in AOD use, but also of GBV, mental health needs, gender, and other social determinants, and how these intersect to affect health outcomes.

Multiple intersecting issues, including the syndemics of gender violence, HIV/STIs, and sexual risk as well as mental health comorbidities and psychological trauma arising from exposure to violence, are highly prevalent among many of the targeted study populations [34,108,109,110]. This review is important because it highlights the need to include HIV and target gender issues in interventions. Although South Africa had the most interventions tested, it is also where more people are living with HIV than anywhere in the world [12,111]. Some interventions have advanced from being purely behavioral to biobehavioral, as the rollout of HIV medication has been successful for treatment and treatment as prevention [112]. As science builds on itself, there are ongoing advancements in the measures available for AOD use; understanding of context, gender differences, and cultural nuances; and on-the-ground implementation. Focusing on AOD alone may not lead to durability of outcomes without addressing the intersectional issues that underpin AOD use. As noted, only the larger randomized trials with formative phases mentioned adaptations to culture and gender within their interventions.

The importance of and ability to integrate services for AOD use with HIV and other chronic disease, such as TB, and antenatal and emergency care is essential [113]. People who use AODs often perceive stigma and experience structural barriers to accessing healthcare [114]. Consequently, early intervention reduces these barriers to care and takes pressure off the overwhelmed AOD treatment system [115]. Further evidence from this review suggests which comprehensive and brief interventions have promising effects. Opportunities for scaling up, adoption, and sustained implementation could realize the promises of these interventions; however, this will take years of commitment from local healthcare systems [28,116].

As noted, several interventions were delivered by peers within their communities, and this review of trials shows that this type of delivery is not only efficient but may increase the intervention’s credibility and acceptance. Brief interventions consisting of one session do not appear to have a great impact, whereas the more comprehensive the intervention, the greater the effect.

Applied researchers may be most comfortable implementing individual or group interventions, as we found few structural interventions. Further, there was a concentrated effort in only a few African countries [117]. Consequently, it is timely for more clinic-level interventions addressing stigma around people who use AODs and community-level interventions for destigmatizing people who use AODs and are living with HIV and to help support the rollout of HIV PrEP for prevention for those at risk. Barriers that women may face when accessing treatment are often different than the barriers men may face. Consequently, sensitivity is needed to the unique needs of underserved populations, along with viewing these needs through a gender lens so that people who use AODs do not feel stigmatized when attempting to access health services [118,119].

Applied research and the impact it has can change policy and ultimately practice. As the world is pushing a social justice agenda, Africa, with its deep patriarchal roots, is lagging in gender equality. Yet, Africa also has some of the most innovative strategists and thought leaders committed to making change within their communities. For example, the first human-to-human heart transplant was in Cape Town, South Africa, by Dr. Christiaan Barnard, a South African surgeon [120].

Despite challenges, it is important to celebrate the achievements of AOD use treatment and prevention efforts in several African countries. For example, there are innovative treatment programs in resource-poor areas, such as the treatment camp approach in Uganda [121]. However, not all of these programs have been rigorously evaluated. Epidemiological evidence has been instrumental in developing formative and pilot studies. These stages are necessary for testing these intervention approaches in RCTs for proving efficacy and effectiveness. Now science is progressing to implementation science to support sustained implementation of evidence-based treatment approaches that improve outcomes [28]. However, one serious limitation among the studies was the lack of interventions addressing injecting drug use beyond exploratory and qualitative studies, as opiate use is continuing to increase in Africa, along with intersectional HIV risk [122]. Within this review, we also recognize sample size limitations that reduce power and the ability to demonstrate significant outcomes over longer follow-up periods. Notably, one project faced a reduced follow-up sample because of COVID-19 [93].

However, several examples of lasting change have been translations of the research focused on AODs and other outcomes into practice. For example, the MATRIX program was first implemented in Cape Town in 2007 and is now provided at many substance use treatment facilities [123,124]. The Women’s Health CoOp has governmental commitments to ongoing training and implementation as part of the usual care treatment system [125,126]. The Teachable Moments Programme was also implemented in South African emergency centers, having been developed as an extension of Sorsdahl et al., 2015 SBIRT [79,127]. However, more implementation data on cost-effectiveness analysis and economic evaluations are needed to demonstrate life cost savings and build an economic case for investment in these interventions [128]. Focus on both and PrEP among couples where AOD-impaired sex is a targeted behavior will be essential to ending the HIV epidemic. These combination interventions can reach people within their communities while also considering community-focused strategies to help with the de-stigmatization of people who use AODs and to reduce their risk of HIV.

A growing body of research is also evaluating implementation science outcomes of AOD use prevention and treatment interventions that address many of the intersectional topics mentioned herein. Confidence and commitment to implementing these kinds of programs is imperative for the long-term sustainability of interventions, which includes having ongoing support from management and higher-level staff, having financial resources, and providing support for clinic staff [126].

## 5. Conclusions

Identifying and addressing AOD use within the context of social determinants is the key to ensuring the long-term health and well-being of people in Africa. The lack of RCT studies addressing injecting drug use in Africa and RCT studies in Northern Africa indicates a need to expand this science further. Additionally, ensuring gender and cultural sensitivity of AOD interventions is essential for optimizing impact. Addressing barriers to treatment and stigma around AOD use and other coexisting conditions, such as HIV, from a structural level at the clinic and community levels is the next frontier, as both are important areas to examine for healthy outcomes and healthy communities.

## Figures and Tables

**Table 1:** Comprehensive interventions targeting individuals who use alcohol and other drugs.

Citation	Region/Sample	Setting/Topics	Intervention	Outcomes
Behavioral				
L’Engle et al. (2014) [70]	**Kenya:** 818 women who conduct sex work living with or at risk for contracting HIV who scored between 7 and 19 on the AUDIT	Six 20-min counseling sessions delivered one-on-one by trained nurses over 6 months at project site EtOH, GBV, SRH	Adapted WHO Brief Motivational Intervention for Alcohol Use and counseling sessions	* Significant reduction in binge drinking at 6- and 12-month follow-ups with intervention participants reporting less than one-third the odds of higher levels of drinking than the control group
Madhombiro et al. (2020) [71]	**Zimbabwe**: 529 adults living with HIV who also had an AUD and were on combination ART	Ten sessions lasting 45 to 60 min each with trained nurses at clinic locations EtOH, HIV	MI-CBT working with participants’ AUDIT scores, personalized information on addressing alcohol use and achieving HIV outcomes	* Difference in AUDIT scores between arms * Reduction in viral load between arms
Murray et al. (2020) [72]	**Zambia:** 123 heterosexual couples where the woman reported experiencing IPV by her male partner	Two-session sex-stratified groups with one additional spousal substance use support session delivered by lay counselors EtOH, IPV	CETA to teach CBT decision rules with elements of psychoeducation and substance use reduction	* Reduction in female reports of past-year IPV and in male reports of perpetrated violence * Significant treatment effects on male alcohol use and smaller effects on female alcohol use
Omeje et al. (2018) [73]	**Nigeria:** 124 community-dwelling adults living with HIV who experience AUD symptoms	20 sessions (50 min each) twice a week, with 2 weeks of follow-up by trained researchers EtOH, HIV	REHT for AUD focused on beliefs related to AUD and techniques to reduce symptoms	* The REHT intervention led to a reduction in AUD symptoms in the treatment group compared with the waitlist control group * Difference in decrease in alcohol-related irrational beliefs between treatment and control groups
Papas et al. (2021) [74]	**Kenya:** 614 adults enrolled as an AMPATH HIV outpatient with hazardous drinking	Six weekly 90-min group sessions delivered by paraprofessional counselors EtOH, HIV	CBT intervention consisting of group sessions teaching coping skills for alcohol reduction	* Significantly lower percentage of drinking days and drinks per drinking day in CBT than healthy life-styles education overall and at all study phases
Wechsberg et al. (2021) [28]	**South Africa:** 480 women living with HIV who used AODs	Implementation science four-cycle stepped- wedge design of an evidence-based intervention delivered by trained clinic staff and researchers in groups at clinics and substance use rehabilitation and treatment centers EtOH, METH, OPI, HIV, IPV/GBV, SRH	Adapted WHC, an evidence-based gender-focused HIV intervention for women living with HIV, to reduce AOD use, GBV, and sexual risk and to increase linkage to HIV care among women who use AODs	* WHC increased ART adherence and reduced alcohol use * Compared with the first cycle, women in the fourth cycle were less likely to report AUD risk and were 4 times more likely to report ART adherence at 6-month follow-up
Wechsberg et al. (2019) [75]	**South Africa:** 641 Black women from 14 mapped AOD “hot spots”	Two intervention group sessions 1 week apart administered by experienced, multilingual female interventionists from the community EtOH, COC, OPI, THC, HIV, SRH, IPV	WHC+, an empowerment-based woman-focused HIV intervention to reduce sex risk, AOD use, and GBV and to increase linkage to HIV care among women who use AODs	* The WHC+ arm was less likely to report frequent heavy drinking and fewer heavy drinking days at 6-month follow-up but not at 12-month follow-up * The WHC+ arm was less likely to report being attacked with a weapon, beaten, or sexually abused by a boyfriend at 6-month follow-up * The WHC+ arm reported more frequent condom negotiation, using a condom while high, and refusing sex without a condom with a boyfriend in the past 3 months at 6-month follow-up
Wechsberg et al. (2016) [76]	**South Africa:** 300 couples: 300 men who used alcohol and their partners (300 women)	3-h workshop delivered by peer educators in groups of couple dyads EtOH, METH, THC, COC, OPI, Methaqualone, GBV, HIV, SRH	CHC adapted from the WHC to provide couples with skill-building exercises around communication and sexual expectations	* Heavy drinking decreased among women in the CHC Heavy drinking decreased among men in all arms Condom use increased in all arms for women; men in the CHC were more likely to report consistent condom use
Wechsberg et al. (2013) [77]	**South Africa:** 720 women who use AODs	Two-session peer-facilitated group intervention with four modules each EtOH, METH, OPI, COC, THC, Methaqualone, GBV, HIV	Adapted WHC, an empowerment-based, woman-focused HIV intervention to reduce sex risk, AOD use, and GBV	At 6-month follow-up: – Women in the WHC arm were more likely to be sober at the last sex than women in the Nutrition arm – More women in the WHC arm were abstinent from drugs as compared with the control group
Wechsberg et al. (2011) [78]	**South Africa:** 617 women who conduct sex work or engage in condomless sex and report alcohol use	Two-session (50 min each) cue card intervention delivered by trained interventionists in groups EtOH, COC, THC, OPI, AMP, METH, Ecstasy, HIV, GBV	WHC, an empowerment-based, woman-focused HIV intervention to reduce sex risk, AOD use, and GBV	Women who did not conduct sex work had a lower mean number of days drinking and were less likely to qualify for alcohol dependence compared with the control group * Greater reductions in drug use among WHC participants * Women who conducted sex work were less likely to report physical abuse by main partner at 6-month follow-up

**Abbreviations:** AMP = amphetamines; AOD = alcohol and other drugs; ART = antiretroviral therapy; AUDIT = Alcohol Use Disorders Identification Test; CBT = cognitive behavioral therapy; CES-D = Center for Epidemiologic Studies Depression Scale; CETA = Common Elements Treatment Approach; CHC = Couples Health CoOp; COC = cocaine; EtOH = alcohol; GBV = gender-based violence; IPV = intimate partner violence; METH = methamphetamines; MI = Motivational Interviewing; OPI = opiates; REHT = Rational emotive health therapy treatment; SRH = sexual and reproductive health; THC = cannabis; WHC = Women’s Health CoOp; WHC+ = Women’s Health CoOp Plus; WHO = World Health Organization. * **Denotes significant finding**.

## Data Availability

This is a review paper. All manuscripts have been published previously.

## Abbreviations

AOD	alcohol and other drugs
ART	antiretroviral therapy
AUD	Alcohol Use Disorder
AUDIT	Alcohol Use Disorders Identification Test
CBT	cognitive behavioral therapy
CES-D	Center for Epidemiologic Studies Depression Scale
CETA	Common Elements Treatment Approach
CHC	Couples Health CoOp
FASD	Fetal Alcohol Spectrum Disorder
GBV	gender-based violence
IMB	Information–Motivation–Behavioral Skills Model
IPV	intimate partner violence
MH	mental health
MI	Motivational Interviewing
MI + PST	Motivational Interviewing and Problem Solving Therapy
PLWH	people living with HIV
PrEP	pre-exposure prophylaxis
RCT	randomized control trial
REHT	Rational emotive health therapy treatment
SBIRTs	screening brief intervention and referral to treatments
SRH	sexual and reproductive health
STI	sexually transmitted infection
TB	tuberculosis
WHC	Women’s Health CoOp
WHC+	Women’s Health CoOp Plus
WHO	World Health Organization

## Appendix A. Search Terms

**Search 1**
(((behavioral) OR (structural)) AND ((intervention) OR (program) OR (project)) AND (Africa) AND ((“substance use disorder”) OR (“substance use”) OR (alcohol) OR (alcoholism) OR (“drug use”) OR (“drug abuse”)) NOT ((“cross-sectional”) OR (review) OR (systematic))) Filters: from January 2010–October 2021
**Search 2**
(((behavioral) OR (structural)) AND ((intervention) OR (program) OR (project) OR (treatment)) AND (Africa) AND ((“substance use disorder”) OR (“substance use”) OR (alcohol) OR (alcoholism) OR (“drug use”) OR (“drug abuse”) OR (methadone) OR (buprenorphine)) NOT ((“cross-sectional”) OR (review) OR (systematic))) Filters: from November 2021–May 2022

## References

[1] Sudhinaraset M., Wigglesworth C., Takeuchi D.T. (2015). Social and cultural contexts of alcohol use. Alcohol Res. Curr. Rev..

[2] National Institute on Drug Abuse (2020). Alcohol. https://www.drugabuse.gov/drug-topics/alcohol#Ref.

[3] United Nations Office on Drugs and Crime (2020). Press Release: UNODC World Drug Report 2020: Global Drug Use Rising; While COVID-19 Has Far Reaching Impact on Global Drug Markets. https://www.unodc.org/unodc/press/releases/2020/June/media-advisory---global-launch-of-the-2020-world-drug-report.html.

[4] Johnson B. (2007). Illicit Drug Markets in South Africa: A Review. https://www.drugabuse.gov/international/abstracts/illicit-drug-markets-in-south-africa-review.

[5] Ferreira-Borges C., Parry C.D.H., Babor T.F. (2017). Harmful use of alcohol: A shadow over Sub-Saharan Africa in need of workable solutions. Int. J. Environ. Res. Public Health.

[6] Eligh J. (2021). A Synthetic Age: The Evolution of Methamphetamine Markets in Eastern and Southern Africa. https://globalinitiative.net/wp-content/uploads/2021/03/GITOC-A-Synthetic-Age-The-Evolution-of-Methamphetamine-Markets-in-Eastern-and-Southern-Africa.pdf.

[7] Statista (2019). Percentage of Illicit Drug Use in Global Population by Drug Type 2019. https://www.statista.com/statistics/443460/percentage-of-population-that-has-used-illicit-drugs-by-drug/.

[8] Donnenfeld Z., Bellow-Schünemann J., Welborn L. (2019). Drug Demand and Use in Africa: Modelling Trends to 2050. https://enact-africa.s3.amazonaws.com/site/uploads/2019-08-28-drug-demand-and-use-in-africa-reaseach-paper-cb.pdf.

[9] South African Community Epidemiology Network on Drug Use January–June 2020 Phase 48: Monitoring Alcohol, Tobacco, and Other Drug Abuse Treatment Admissions in South Africa. https://www.samrc.ac.za/sites/default/files/attachments/2021-07-29/SACENDU%20Full%20Report_Phase%2048_July%202020.pdf.

[10] Wechsberg W.M., Jones H.E., Zule W.A., Myers B.J., Browne F.A., Kaufman M.R., Luseno W., Flisher A.J., Parry C.D. (2010). Methamphetamine (“tik”) use and its association with condom use among out-of-school females in Cape Town, South Africa. Am. J. Drug Alcohol Abuse.

[11] UNAIDS (2021). UNAIDS Fact Sheet 2021: Global HIV Statistics. https://www.unaids.org/en/resources/fact-sheet.

[12] UNAIDS (2020). AIDSinfo: Global Data on HIV Epidemiology and Response. http://aidsinfo.unaids.org/.

[13] United Nations Office on Drugs and Crime (2018). World Drug Report: Women and Drugs. https://www.unodc.org/wdr2018/prelaunch/WDR18_Booklet_5_WOMEN.pdf.

[14] United Nations Office on Drugs and Crime (2021). World Drug Report. https://www.unodc.org/unodc/data-and-analysis/wdr2021.html.

[15] World Health Organization (2018). Fact Sheet: Alcohol. https://www.who.int/news-room/fact-sheets/detail/alcohol.

[16] Tapert S.F., Caldwell L., Burke C. (2004). Alcohol and the adolescent brain: Human studies. Alcohol Res Health.

[17] Salmanzadeh H., Ahmadi-Soleimani S.M., Pachenari N., Azadi M., Halliwell R.F., Rubino T., Azizi H. (2020). Adolescent drug exposure: A review of evidence for the development of persistent changes in brain function. Brain Res. Bull.

[18] Raiford J.L., Herbst J.H., Carry M., Browne F.A., Doherty I., Wechsberg W.M. (2014). Low prospects and high risk: Structural determinants of health associated with sexual risk among young African American women residing in resource-poor communities in the south. Am. J. Community Psychol.

[19] Myers B., Carney T., Johnson K., Browne F.A., Wechsberg W.M. (2020). Service providers’ perceptions of barriers to the implementation of trauma-focused substance use services for women in Cape Town, South Africa. Int. J. Drug Policy.

[20] Meyer J.P., Springer S.A., Altice F.L. (2011). Substance abuse, violence, and HIV in women: A literature review of the syndemic. J. Womens Health.

[21] Bonner C.P., Browne F.A., Ndirangu J.W., Howard B., Zule W.A., Speizer I.S., Kline T., Wechsberg W.M. (2019). Exploring the associations between physical and sexual gender-based violence and HIV among women who use substances in South Africa: The role of agency and alcohol. AIDS Care.

[22] Adebiyi B.O., Mukumbang F.C., Beytell A.-M. (2019). To what extent is Fetal Alcohol Spectrum Disorder considered in policy-related documents in South Africa? A document review. Health Res. Policy Syst.

[23] Ritchie H.R.M. (2019). Our World in Data: Alcohol Consumption. https://ourworldindata.org/alcohol-consumption.

[24] Kaiser Family Foundation (2021). The Global HIV/AIDS Epidemic Fact Sheet. https://www.kff.org/global-health-policy/fact-sheet/the-global-hivaids-epidemic/.

[25] El-Bassel N., Gilbert L., Witte S., Wu E., Gaeta T., Schilling R., Wada T. (2003). Intimate partner violence and substance abuse among minority women receiving care from an inner-city emergency department. Womens Health.

[26] El-Bassel N., Gilbert L., Witte S., Wu E., Chang M. (2011). Intimate partner violence and HIV among drug-involved women: Contexts linking these two epidemics--challenges and implications for prevention and treatment. Subst. Use Misuse.

[27] World Health Organization (2006). WHO Facts on Alcohol and Violence: Intimate Partner Violence and Alcohol. https://www.who.int/violence_injury_prevention/violence/world_report/factsheets/fs_intimate.pdf.

[28] Wechsberg W.M., Browne F.A., Ndirangu J., Bonner C.P., Kline T.L., Gichane M., Zule W.A. (2021). Outcomes of implementing in the real world the Women’s Health CoOp intervention in Cape Town, South Africa. AIDS Behav..

[29] El-Krab R., Kalichman S.C. (2021). Alcohol-antiretroviral therapy interactive toxicity beliefs and intentional medication nonadherence: Review of research with implications for interventions. AIDS Behav..

[30] Rose A.L., Belus J.M., Ma T., Lee J.S., Wan C., De Los Reyes A., Joska J.A., Andersen L.S., Myers B., Magidson J.F. (2022). The relationship between harmful alcohol use and antiretroviral non-adherence in people accessing HIV treatment in Cape Town, South Africa: An event-level analysis. AIDS Behav..

[31] UNAIDS (2019). Global AIDS Update 2019—Communities at the Centre: Defending Rights Breaking Barriers, Reaching People with HIV Services. https://www.unaids.org/sites/default/files/media_asset/2019-global-AIDS-update_en.pdf.

[32] Sileo K.M., Miller A.P., Huynh T.A., Kiene S.M. (2020). A systematic review of interventions for reducing heavy episodic drinking in sub-Saharan African settings. PLoS ONE.

[33] Biggane A.M., Briegal E., Obasi A. (2021). Interventions for adolescent alcohol consumption in Africa: Protocol for a scoping review including an overview of reviews. Syst. Rev.

[34] Wechsberg W.M., Browne F.A., Bonner C.P., Washio Y., Howard B.N., van der Drift I. (2021). Current interventions for people living with HIV who use alcohol: Why gender matters. Curr. HIV/AIDS Rep..

[35] Kalichman S.C., Simbayi L.C., Kaufman M., Cain D., Jooste S. (2007). Alcohol use and sexual risks for HIV/AIDS in sub-Saharan Africa: Systematic review of empirical findings. Prev. Sci..

[36] Tomokawa S., Miyake K., Akiyama T., Makino Y., Nishio A., Kobayashi J., Jimba M., Ayi I., Njenga S.M., Asakura T. (2020). Effective school-based preventive interventions for alcohol use in Africa: A systematic review. Afr. Health Sci..

[37] Wechsberg W.M., Browne F.A., Ndirangu J., Bonner C.P., Minnis A.M., Nyblade L., Speizer I.S., Howard B.N., Myers B., Ahmed K. (2020). The PrEPARE Pretoria Project: Protocol for a cluster-randomized factorial-design trial to prevent HIV with PrEP among adolescent girls and young women in Tshwane, South Africa. BMC Public Health.

[38] Staton C.A., Zadey S., O’Leary P., Phillips A., Minja L., Swahn M.H., Hirshon J.M., Boshe J., Sakita F., Vissoci J.R.N. (2022). PRACT: A pragmatic randomized adaptive clinical trial protocol to investigate a culturally adapted brief negotiational intervention for alcohol use in the emergency department in Tanzania. Trials.

[39] Huis In ‘t Veld D., Skaal L., Peltzer K., Colebunders R., Ndimande J.V., Pengpid S. (2012). The efficacy of a brief intervention to reduce alcohol misuse in patients with HIV in South Africa: Study protocol for a randomized controlled trial. Trials.

[40] Kane J.C., Skavenski Van Wyk S., Murray S.M., Bolton P., Melendez F., Danielson C.K., Chimponda P., Munthali S., Murray L.K. (2017). Testing the effectiveness of a transdiagnostic treatment approach in reducing violence and alcohol abuse among families in Zambia: Study protocol of the Violence and Alcohol Treatment (VATU) trial. Glob. Ment. Health.

[41] Kuo C.C., Sibeko G., Akande M., Allie S., Tisaker N., Stein D.J., Becker S.J. (2021). Advancing a cascading train-the-trainer model of frontline HIV service providers in South Africa: Protocol of an implementation trial. Addict. Sci. Clin. Pract..

[42] Leddy A.M., Hahn J.A., Getahun M., Emenyonu N.I., Woolf-King S.E., Sanyu N., Katusiime A., Fatch R., Chander G., Hutton H.E. (2021). Cultural adaptation of an intervention to reduce hazardous alcohol use among people living with HIV in Southwestern Uganda. AIDS Behav..

[43] Madhombiro M., Dube-Marimbe B., Dube M., Chibanda D., Zunza M., Rusakaniko S., Stewart D., Seedat S. (2017). A cluster randomised controlled trial protocol of an adapted intervention for alcohol use disorders in people living with HIV and AIDS: Impact on alcohol use, general functional ability, quality of life and adherence to HAART. BMC Psychiatry.

[44] Musyoka C.M., Mbwayo A., Donovan D.M., Mathai M. (2021). mHealth-based peer mentoring for prevention of alcohol and substance abuse among first year university students: Protocol for quasi-experimental intervention. J. Subst. Use.

[45] Parry C.D., Morojele N.K., Myers B.J., Kekwaletswe C.T., Manda S.O., Sorsdahl K., Ramjee G., Hahn J.A., Rehm J., Shuper P.A. (2014). Efficacy of an alcohol-focused intervention for improving adherence to antiretroviral therapy (ART) and HIV treatment outcomes—A randomised controlled trial protocol. BMC Infect. Dis..

[46] Rotheram-Borus M.J., Tomlinson M., Mayekiso A., Bantjes J., Harris D.M., Stewart J., Weiss R.E. (2018). Gender-specific HIV and substance abuse prevention strategies for South African men: Study protocol for a randomized controlled trial. Trials.

[47] Tzilos Wernette G., Plegue M., Mmeje O., Sen A., Countryman K., Ngo Q., Prosser L., Zlotnick C. (2019). Reducing sexual health risks and substance use in the prenatal setting: A study protocol for a randomized controlled trial. Contemp. Clin. Trials.

[48] Jewkes R., Gibbs A., Jama-Shai N., Willan S., Misselhorn A., Mushinga M., Washington L., Mbatha N., Skiweyiya Y. (2014). Stepping Stones and Creating Futures intervention: Shortened interrupted time series evaluation of a behavioural and structural health promotion and violence prevention intervention for young people in informal settlements in Durban, South Africa. BMC Public Health.

[49] Sorsdahl K., Myers B., Ward C., Matzopoulos R., Mtukushe B., Nicol A., Stein D.J. (2014). Screening and brief interventions for substance use in emergency departments in the Western Cape province of South Africa: Views of health care professionals. Int. J. Inj. Control Saf. Promot..

[50] Beres L.K., Mbabali I., Anok A., Katabalwa C., Mulamba J., Thomas A.G., Bugos E., Nakigozi G., Grabowski M.K., Chang L.W. (2021). Mobile ecological momentary assessment and intervention and health behavior change among adults in Rakai, Uganda: Pilot randomized controlled trial. JMIR Form Res..

[51] Bhana A., Mellins C.A., Petersen I., Alicea S., Myeza N., Holst H., Abrams E., John S., Chhagan M., Nestadt D.F. (2014). The VUKA family program: Piloting a family-based psychosocial intervention to promote health and mental health among HIV infected early adolescents in South Africa. AIDS Care.

[52] Calligaro G.L., de Wit Z., Cirota J., Orrell C., Myers B., Decker S., Stein D.J., Sorsdahl K., Dawson R. (2021). Brief psychotherapy administered by non-specialised health workers to address risky substance use in patients with multidrug-resistant tuberculosis: A feasibility and acceptability study. Pilot Feasibility Stud..

[53] Carney T., Browne F.A., Myers B., Kline T.L., Howard B., Wechsberg W.M. (2019). Adolescent female school dropouts who use drugs and engage in risky sex: Effects of a brief pilot intervention in Cape Town, South Africa. AIDS Care.

[54] Papas R.K., Sidle J.E., Gakinya B.N., Baliddawa J.B., Martino S., Mwaniki M.M., Songole R., Omolo O.E., Kamanda A.M., Ayuku D.O. (2011). Treatment outcomes of a stage 1 cognitive-behavioral trial to reduce alcohol use among human immunodeficiency virus-infected out-patients in western Kenya. Addiction.

[55] Sorsdahl K., Stein D.J., Weich L., Fourie D., Myers B. (2012). The effectiveness of a hospital-based intervention for patients with substance-use problems in the Western Cape. S. Afr. Med. J..

[56] Carney T., Johnson K., Carrico A., Myers B. (2020). Acceptability and feasibility of a brief substance use intervention for adolescents in Cape Town, South Africa: A pilot study. Int. J. Psychol..

[57] Madhombiro M., Dube B., Dube M., Zunza M., Chibanda D., Rusakaniko S., Seedat S. (2019). Intervention for alcohol use disorders at an HIV care clinic in Harare: A pilot and feasibility study. Addict. Sci. Clin. Pract..

[58] Magidson J.F., Joska J.A., Belus J.M., Andersen L.S., Regenauer K.S., Rose A.L., Myers B., Majokweni S., O’Cleirigh C., Safren S.A. (2021). Project Khanya: Results from a pilot randomized type 1 hybrid effectiveness-implementation trial of a peer-delivered behavioural intervention for ART adherence and substance use in HIV care in South Africa. J. Int. AIDS Soc..

[59] Rotheram-Borus M.J., Lightfoot M., Kasirye R., Desmond K. (2012). Vocational training with HIV prevention for Ugandan youth. AIDS Behav..

[60] Tang A.M., Hamunime N., Adams R.A., Kanyinga G., Fischer-Walker C., Agolory S., Prybylski D., Mutenda N., Sughrue S., Walker D.D. (2019). Introduction of an alcohol-related electronic screening and brief intervention (eSBI) program to reduce hazardous alcohol consumption in Namibia’s antiretroviral treatment (ART) program. AIDS Behav..

[61] Conroy A.A., Ruark A., McKenna S.A., Tan J.Y., Darbes L.A., Hahn J.A., Mkandawire J. (2020). The unaddressed needs of alcohol-using couples on antiretroviral therapy in Malawi: Formative research on multilevel interventions. AIDS Behav..

[62] Kerrigan D., Mbwambo J., Likindikoki S., Beckham S., Mwampashi A., Shembilu C., Mantsios A., Leddy A., Davis W., Galai N. (2017). Project Shikamana: Baseline findings from a community empowerment-based combination HIV prevention trial among female sex workers in Iringa, Tanzania. J. Acquir. Immune Defic. Syndr..

[63] Kane J.C., Sharma A., Murray L.K., Chander G., Kanguya T., Skavenski S., Chitambi C., Lasater M.E., Paul R., Cropsey K. (2022). Efficacy of the Common Elements Treatment Approach (CETA) for unhealthy alcohol use among adults with HIV in Zambia: Results from a pilot randomized controlled trial. AIDS Behav..

[64] Magidson J.F., Andersen L.S., Satinsky E.N., Myers B., Kagee A., Anvari M., Joska J.A. (2020). “Too much boredom isn’t a good thing”: Adapting behavioral activation for substance use in a resource-limited South African HIV care setting. Psychotherapy.

[65] Morojele N.K., Kitleli N., Ngako K., Kekwaletswe C.T., Nkosi S., Fritz K., Parry C.D. (2014). Feasibility and acceptability of a bar-based sexual risk reduction intervention for bar patrons in Tshwane, South Africa. Sahara J..

[66] Myers B., Carney T., Browne F.A., Wechsberg W.M. (2019). A trauma-informed substance use and sexual risk reduction intervention for young South African women: A mixed-methods feasibility study. BMJ Open.

[67] Myers B., Parry C.D.H., Morojele N.K., Nkosi S., Shuper P.A., Kekwaletswe C.T., Sorsdahl K.R. (2020). “Moving forward with life”: Acceptability of a brief alcohol reduction intervention for people receiving antiretroviral therapy in South Africa. Int. J. Environ. Res. Public Health..

[68] Myers B., Sorsdahl K., Morojele N.K., Kekwaletswe C., Shuper P.A., Parry C.D. (2017). “In this thing I have everything I need”: Perceived acceptability of a brief alcohol-focused intervention for people living with HIV. AIDS Care.

[69] Sorsdahl K., Stein D.J., Pasche S., Jacobs Y., Kader R., Odlaug B., Richter S., Myers B., Grant J.E. (2021). A novel brief treatment for methamphetamine use disorders in South Africa: A randomised feasibility trial. Addict. Sci. Clin. Pract..

[70] LʼEngle K.L., Mwarogo P., Kingola N., Sinkele W., Weiner D.H. (2014). A randomized controlled trial of a brief intervention to reduce alcohol use among female sex workers in Mombasa, Kenya. J. Acquir. Immune Defic. Syndr..

[71] Madhombiro M., Kidd M., Dube B., Dube M., Mutsvuke W., Muronzie T., Zhou D.T., Derveeuw S., Chibanda D., Chingono A. (2020). Effectiveness of a psychological intervention delivered by general nurses for alcohol use disorders in people living with HIV in Zimbabwe: A cluster randomized controlled trial. J. Int. AIDS Soc..

[72] Murray L.K., Kane J.C., Glass N., Skavenski van Wyk S., Melendez F., Paul R., Kmett Danielson C., Murray S.M., Mayeya J., Simenda F. (2020). Effectiveness of the Common Elements Treatment Approach (CETA) in reducing intimate partner violence and hazardous alcohol use in Zambia (VATU): A randomized controlled trial. PLoS Med..

[73] Omeje J.C., Otu M.S., Aneke A.O., Adikwu V.O., Nwaubani O.O., Chigbu E.F., Onuigbo L.N., Udom I.E., Aye E.N., Akaneme I.N. (2018). Effect of rational emotive health therapy on alcohol use among community-dwelling, HIV-positive patients. Medicine.

[74] Papas R.K., Gakinya B.N., Mwaniki M.M., Lee H., Keter A.K., Martino S., Klein D.A., Liu T., Loxley M.P., Sidle J.E. (2021). A randomized clinical trial of a group cognitive-behavioral therapy to reduce alcohol use among human immunodeficiency virus-infected outpatients in western Kenya. Addiction.

[75] Wechsberg W.M., Bonner C.P., Zule W.A., van der Horst C., Ndirangu J., Browne F.A., Kline T.L., Howard B.N., Rodman N.F. (2019). Addressing the nexus of risk: Biobehavioral outcomes from a cluster randomized trial of the Women’s Health CoOp Plus in Pretoria, South Africa. Drug Alcohol. Depend..

[76] Wechsberg W.M., Zule W.A., El-Bassel N., Doherty I.A., Minnis A.M., Novak S.D., Myers B., Carney T. (2016). The male factor: Outcomes from a cluster randomized field experiment with a couples-based HIV prevention intervention in a South African township. Drug Alcohol Depend..

[77] Wechsberg W.M., Jewkes R., Novak S.P., Kline T., Myers B., Browne F.A., Carney T., Morgan Lopez A.A., Parry C. (2013). A brief intervention for drug use, sexual risk behaviours and violence prevention with vulnerable women in South Africa: A randomised trial of the Women’s Health CoOp. BMJ Open.

[78] Wechsberg W.M., Zule W.A., Luseno W.K., Kline T.L., Browne F.A., Novak S.P., Ellerson R.M. (2011). Effectiveness of an adapted evidence-based woman-focused intervention for sex workers and non-sex workers: The Women’s Health CoOp in South Africa. J. Drug Issues.

[79] Sorsdahl K., Stein D.J., Corrigall J., Cuijpers P., Smits N., Naledi T., Myers B. (2015). The efficacy of a blended motivational interviewing and problem solving therapy intervention to reduce substance use among patients presenting for emergency services in South Africa: A randomized controlled trial. Subst. Abuse Treat. Prev. Policy.

[80] Harder V.S., Musau A.M., Musyimi C.W., Ndetei D.M., Mutiso V.N. (2020). A randomized clinical trial of mobile phone motivational interviewing for alcohol use problems in Kenya. Addiction.

[81] Huis In ‘t Veld D., Ensoy-Musoro C., Pengpid S., Peltzer K., Colebunders R. (2019). The efficacy of a brief intervention to reduce alcohol use in persons with HIV in South Africa, a randomized clinical trial. PLoS ONE.

[82] Rendall-Mkosi K., Morojele N., London L., Moodley S., Singh C., Girdler-Brown B. (2013). A randomized controlled trial of motivational interviewing to prevent risk for an alcohol-exposed pregnancy in the Western Cape, South Africa. Addiction.

[83] Wandera B., Tumwesigye N.M., Nankabirwa J.I., Mafigiri D.K., Parkes-Ratanshi R.M., Kapiga S., Hahn J., Sethi A.K. (2017). Efficacy of a single, brief alcohol reduction intervention among men and women living with HIV/AIDS and using alcohol in Kampala, Uganda: A randomized trial. J. Int. Assoc. Provid. AIDS Care.

[84] Pengpid S., Peltzer K., Van der Heever H., Skaal L. (2013). Screening and Brief Interventions for Hazardous and Harmful Alcohol Use among University Students in South Africa: Results from a Randomized Controlled Trial. Int. J. Environ. Res. Public Health.

[85] Mertens J.R., Ward C.L., Bresick G.F., Broder T., Weisner C.M. (2014). Effectiveness of nurse-practitioner-delivered brief motivational intervention for young adult alcohol and drug use in primary care in South Africa: A randomized clinical trial. Alcohol Alcohol..

[86] Marais S., Jordaan E., Viljoen D., Olivier L., de Waal J., Poole C. (2011). The effect of brief interventions on the drinking behaviour of pregnant women in a high-risk rural South African community: A cluster randomised trial. Early Child Dev. Care.

[87] Ward C.L., Mertens J.R., Bresick G.F., Little F., Weisner C.M. (2015). Screening and brief intervention for substance misuse: Does it reduce aggression and HIV-related risk behaviours?. Alcohol Alcohol..

[88] Peltzer K., Naidoo P., Louw J., Matseke G., Zuma K., McHunu G., Tutshana B., Mabaso M. (2013). Screening and brief interventions for hazardous and harmful alcohol use among patients with active tuberculosis attending primary public care clinics in South Africa: Results from a cluster randomized controlled trial. BMC Public Health.

[89] Pengpid S., Peltzer K., Skaal L., Van der Heever H. (2013). Screening and brief interventions for hazardous and harmful alcohol use among hospital outpatients in South Africa: Results from a randomized controlled trial. BMC Public Health.

[90] Rotheram-Borus M.J., Tomlinson M., Le Roux I., Stein J.A. (2015). Alcohol Use, Partner Violence, and Depression A Cluster Randomized Controlled Trial Among Urban South African Mothers Over 3 Years. Am. J. Prev. Med..

[91] Peltzer K., Tabane C., Matseke G., Simbayi L. (2010). Lay counsellor-based risk reduction intervention with HIV positive diagnosed patients at public HIV counselling and testing sites in Mpumalanga, South Africa. Eval. Program Plan..

[92] Pitpitan E.V., Kalichman S.C., Garcia R.L., Cain D., Eaton L.A., Simbayi L.C. (2015). Mediators of behavior change resulting from a sexual risk reduction intervention for STI patients, Cape Town, South Africa. J. Behav. Med..

[93] Louwagie G., Kanaan M., Morojele N.K., Van Zyl A., Moriarty A.S., Li J., Siddiqi K., Turner A., Mdege N.D., Omole O.B. (2022). Effect of a brief motivational interview and text message intervention targeting tobacco smoking, alcohol use and medication adherence to improve tuberculosis treatment outcomes in adult patients with tuberculosis: A multicentre, randomised controlled trial of the ProLife programme in South Africa. BMJ Open.

[94] Jemmott III J.B., Jemmott L.S., O’Leary A., Ngwane Z., Icard L.D., Heeren G.A., Mtose X., Carty C. (2014). Cluster-randomized controlled trial of an HIV/sexually transmitted infection risk-reduction intervention for South African men. Am. J. Public Health.

[95] Tibbits M.K., Smith E.A., Caldwell L.L., Flisher A.J. (2011). Impact of HealthWise South Africa on polydrug use and high-risk sexual behavior. Health Educ. Res.

[96] Manyaapelo T., Van den Borne B., Ruiter R.A.C., Sifunda S., Reddy P. (2019). Effectiveness of a health behavioural intervention aimed at reduction of risky sexual behaviours among young men in the KwaZulu-Natal province, South Africa. Int. J. Environ. Res. Public Health.

[97] Moscoe E., Agot K., Thirumurthy H. (2019). Effect of a prize-linked savings intervention on savings and healthy behaviors among Mmen in Kenya: A randomized clinical trial. JAMA Netw. Open.

[98] Massarwi A.A., Cluver L., Meinck F., Doubt J., Lachman J.M., Shenderovich Y., Green O. (2021). Mediation pathways for reduced substance use among parents in South Africa: A randomized controlled trial. BMC Public Health.

[99] Eze I.I., Mbachu C.O., Azuogu B.N., Ossai E., Unah A.I., Akamike I.C., Onwasigwe C.N. (2021). Effect of on-site behavioural modification intervention on lifestyle risk factors of hypertension among adult market traders in Abakaliki, Nigeria. Int. J. Health Promot. Educ..

[100] Bachanas P., Kidder D., Medley A., Pals S.L., Carpenter D., Howard A., Antelman G., DeLuca N., Muhenje O., Sheriff M. (2016). Delivering prevention interventions to people living with HIV in clinical care settings: Results of a cluster randomized trial in Kenya, Namibia, and Tanzania. AIDS Behav..

[101] Burnhams N.H., London L., Laubscher R., Nel E., Parry C. (2015). Results of a cluster randomised controlled trial to reduce risky use of alcohol, alcohol-related HIV risks and improve help-seeking behaviour among safety and security employees in the Western Cape, South Africa. Subst. Abuse Treat. Prev. Policy.

[102] Cubbins L.A., Kasprzyk D., Montano D., Jordan L.P., Woelk G. (2012). Alcohol use and abuse among rural Zimbabwean adults: A test of a community-level intervention. Drug Alcohol Depend..

[103] Wagman J.A., Gray R.H., Campbell J.C., Thoma M., Ndyanabo A., Ssekasanvu J., Nalugoda F., Kagaayi J., Nakigozi G., Serwadda D. (2015). Effectiveness of an integrated intimate partner violence and HIV prevention intervention in Rakai, Uganda: Analysis of an intervention in an existing cluster randomised cohort. Lancet Glob. Health.

[104] London L. (1999). The dop’system, alcohol abuse and social control amongst farm workers in South Africa: A public health challenge. Soc. Sci. Med.

[105] Martinez P., Røislien J., Naidoo N., Clausen T. (2011). Alcohol abstinence and drinking among African women: Data from the World Health Surveys. BMC Public Health.

[106] Lange S., Probst C., Gmel G., Rehm J., Burd L., Popova S. (2017). Global prevalence of Fetal Alcohol Spectrum Disorder among children and youth: A systematic review and meta-analysis. JAMA Pediatr..

[107] Adnams C.M. (2017). Fetal alcohol spectrum disorder in Africa. Curr. Opin. Psychiatry.

[108] Myers B., Joska J.A., Lund C., Levitt N.S., Butler C.C., Naledi T., Milligan P., Stein D.J., Sorsdahl K. (2018). Patient preferences for the integration of mental health counseling and chronic disease care in South Africa. Patient Prefer. Adherence.

[109] Abrahams N., Mhlongo S., Chirwa E., Lombard C., Dunkle K., Seedat S., Kengne A.P., Myers B., Peer N., García-Moreno C.M. (2020). Rape survivors in South Africa: Analysis of the baseline socio-demographic and health characteristics of a rape cohort. Glob. Health Action.

[110] Myers B., Bantjes J., Lochner C., Mortier P., Kessler R.C., Stein D.J. (2021). Maltreatment during childhood and risk for common mental disorders among first year university students in South Africa. Soc. Psychiatry Psychiatr. Epidemiol.

[111] Statistics South Africa (2021). Mid-Year Population Estimates. https://www.statssa.gov.za/publications/P0302/P03022021.pdf.

[112] Wechsberg W.M., van der Horst C., Ndirangu J., Doherty I.A., Kline T., Browne F.A., Belus J.M., Nance R., Zule W.A. (2017). Seek, test, treat: Substance-using women in the HIV treatment cascade in South Africa. Addict. Sci. Clin. Pract..

[113] Myers B., Lund C., Lombard C., Joska J., Levitt N., Butler C., Cleary S., Naledi T., Milligan P., Stein D.J. (2018). Comparing dedicated and designated models of integrating mental health into chronic disease care: Study protocol for a cluster randomized controlled trial. Trials.

[114] Howard B.N., Van Dorn R., Myers B.J., Zule W.A., Browne F.A., Carney T., Wechsberg W.M. (2017). Barriers and facilitators to implementing an evidence-based woman-focused intervention in South African health services. BMC Health Serv. Res.

[115] Myers B., Kline T.L., Doherty I.A., Carney T., Wechsberg W.M. (2014). Perceived need for substance use treatment among young women from disadvantaged communities in Cape Town, South Africa. BMC Psychiatry.

[116] Morris Z.S., Wooding S., Grant J. (2011). The answer is 17 years, what is the question: Understanding time lags in translational research. J. R. Soc. Med.

[117] Jacobs Y., Myers B., van der Westhuizen C., Brooke-Sumner C., Sorsdahl K. (2021). Task sharing or task dumping: Counsellors experiences of delivering a psychosocial intervention for mental health problems in South Africa. Community Ment. Health J..

[118] Myers B., Louw J., Pasche S. (2011). Gender differences in barriers to alcohol and other drug treatment in Cape Town South Africa. Afr. J. Psychiatry.

[119] Myers B., Carney T., Wechsberg W.M. (2016). “Not on the agenda”: A qualitative study of influences on health services use among poor young women who use drugs in Cape Town, South Africa. Int. J. Drug Policy.

[120] Cooper D.K. (2018). Christiaan Barnard—The surgeon who dared: The story of the first human-to-human heart transplant. Glob. Cardiol. Sci. Pract..

[121] Ertl V., Groß M., Mwaka S.O., Neuner F. (2021). Treating alcohol use disorder in the absence of specialized services—Evaluation of the moving inpatient Treatment Camp approach in Uganda. BMC Psychiatry.

[122] Kurth A.E., Cherutich P., Conover R., Chhun N., Bruce R.D., Lambdin B.H. (2018). The opioid epidemic in Africa and its impact. Curr. Addict. Rep..

[123] Gouse H. Implementation of the Matrix Model in Cape Town, South Africa: Evaluating treatment engagement and relapse outcomes. Proceedings of the 2015 College on Problems of Drug Dependence.

[124] Huber A., Ling W., Shoptaw S., Gulati V., Brethen P., Rawson R. (1997). Integrating treatments for methamphetamine abuse: A psychosocial perspective. J. Addict. Dis..

[125] Gichane M.W., Wechsberg W.M., Ndirangu J., Browne F.A., Bonner C.P., Grimwood A., Shaikh N., Howard B., Zule W.A. (2020). Implementation science outcomes of a gender-focused HIV and alcohol risk-reduction intervention in usual-care settings in South Africa. Drug Alcohol Depend..

[126] Gichane M.W., Wechsberg W.M., Ndirangu J., Howard B., Bonner C.P., Browne F.A., Zule W.A. (2021). Sustainability of a gender-focused HIV and alcohol risk-reduction intervention in usual care settings in South Africa: A mixed methods analysis. AIDS Care.

[127] van der Westhuizen C., Myers B., Malan M., Naledi T., Roelofse M., Stein D.J., Lahri S.a., Sorsdahl K. (2019). Implementation of a screening, brief intervention and referral to treatment programme for risky substance use in South African emergency centres: A mixed methods evaluation study. PLoS ONE.

[128] Mutyambizi-Mafunda V., Myers B., Sorsdahl K., Chanakira E., Lund C., Cleary S. (2021). Economic evaluations of psychological treatments for common mental disorders in low-and middle-income countries: Protocol for a systematic review. Glob. Health Action.

